# Environmental and health impacts of spraying COVID-19 disinfectants with associated challenges

**DOI:** 10.1007/s11356-021-16575-7

**Published:** 2021-10-01

**Authors:** Shakeel Ahmad Bhat, Farooq Sher, Rohitashw Kumar, Emina Karahmet, Syed Anam Ul Haq, Ayesha Zafar, Eder C. Lima

**Affiliations:** 1 College of Agricultural Engineering, Sher-e-Kashmir University of Agricultural Sciences and Technology of Kashmir, Jammu and Kashmir, India; 2 Department of Engineering, School of Science and Technology, Nottingham Trent University, Nottingham, NG11 8NS UK; 3 Department of Biochemistry, Faculty of Pharmacy, University of Modern Science, 88000 Mostar, Bosnia and Herzegovina; 4 International Society of Engineering Science and Technology, Nottingham, UK; 5 Division of Plant Biotechnology, Sher-e-Kashmir University of Agricultural Sciences and Technology of Kashmir, Srinagar, Jammu and Kashmir 190025 India; 6 Institute of Biochemistry and Biotechnology, Faculty of Biosciences, University of Veterinary and Animal Sciences, Lahore, Pakistan; 7 Institute of Chemistry, Federal University of Rio Grande do Sul (UFRGS), Av. Bento Goncalves 9500, P.O. Box 15003, Porto Alegre, RS ZIP 91501-970 Brazil

**Keywords:** Environmental pollution, Health impacts, Disinfectant, Chemicals, Pandemic, Pollution and COVID-19

## Abstract

Coronavirus refers to a group of widespread viruses. The name refers to the specific morphology of these viruses because their spikes look like a crown under an electron microscope. The outbreak of coronavirus disease 2019 (COVID-19) that has been reported in Wuhan, China, in December 2019, was proclaimed an international public health emergency (PHEIC) on 30 January 2020, and on 11 March 2020, it was declared as a pandemic (World Health Organization 2020). The official name of the virus was declared by the WHO as “COVID-19 virus”, formerly known as “2019-nCoV”, or “Wuhan Coronavirus”. The International Committee on Virus Taxonomy’s Coronavirus Research Group has identified that this virus is a form of coronavirus that caused a severe outbreak of acute respiratory syndrome in 2002–2003 (SARS). As a result, the latest severe acute respiratory syndrome has been classified as a corona virus 2 (SARS-CoV-2) pathogen by this committee. This disease spread quickly across the country and the world within the first 3 months of the outbreak and became a global pandemic. To stop COVID-19 from spreading, the governing agencies used various chemicals to disinfect different commercial spaces, streets and highways. However, people used it aggressively because of panic conditions, anxiety and unconsciousness, which can have a detrimental impact on human health and the environment. Our water bodies, soil and air have been polluted by disinfectants, forming secondary products that can be poisonous and mutagenic. In the prevention and spread of COVID-19, disinfection is crucial, but disinfection should be carried out with sufficient precautions to minimize exposure to harmful by-products. In addition, to prevent inhalation, adequate personal protective equipment should be worn and chemical usage, concentrations, ventilation in the room and application techniques should be carefully considered. In the USA, 60% of respondents said they cleaned or disinfected their homes more often than they had in the previous months. In addition to the robust use of disinfection approaches to combat COVID-19, we will explore safe preventative solutions here.

## Introduction

Coronavirus is a term that refers to a category of viruses that are quite common. The name alludes to the viruses’ unique appearance, which includes spikes on their surface that resemble a crown under an electron microscope. Coronaviruses are a wide family of viruses that infect animals and humans, causing illnesses ranging from colds to more serious infections like Middle East respiratory syndrome (MERS-CoV) and acute respiratory syndrome (SARS-CoV)(Al Hajjar et al. 2013; Chan et al. 2015; Mohd et al. 2016). The outbreak of a novel coronavirus disease (2019-nCoV) that started in Wuhan, China, has now spread to 26 countries around the world (Bhat et al. 2021). India has one of the lowest COVID-19 fatality rates in the world (2.8% against a global average of 4.7%). Furthermore, Indians have been found to have a considerably higher recovery rate than non-Indians (60.9% versus a global average of 56.6% ) (Samaddar et al. 2020).

**Fig. 1:**
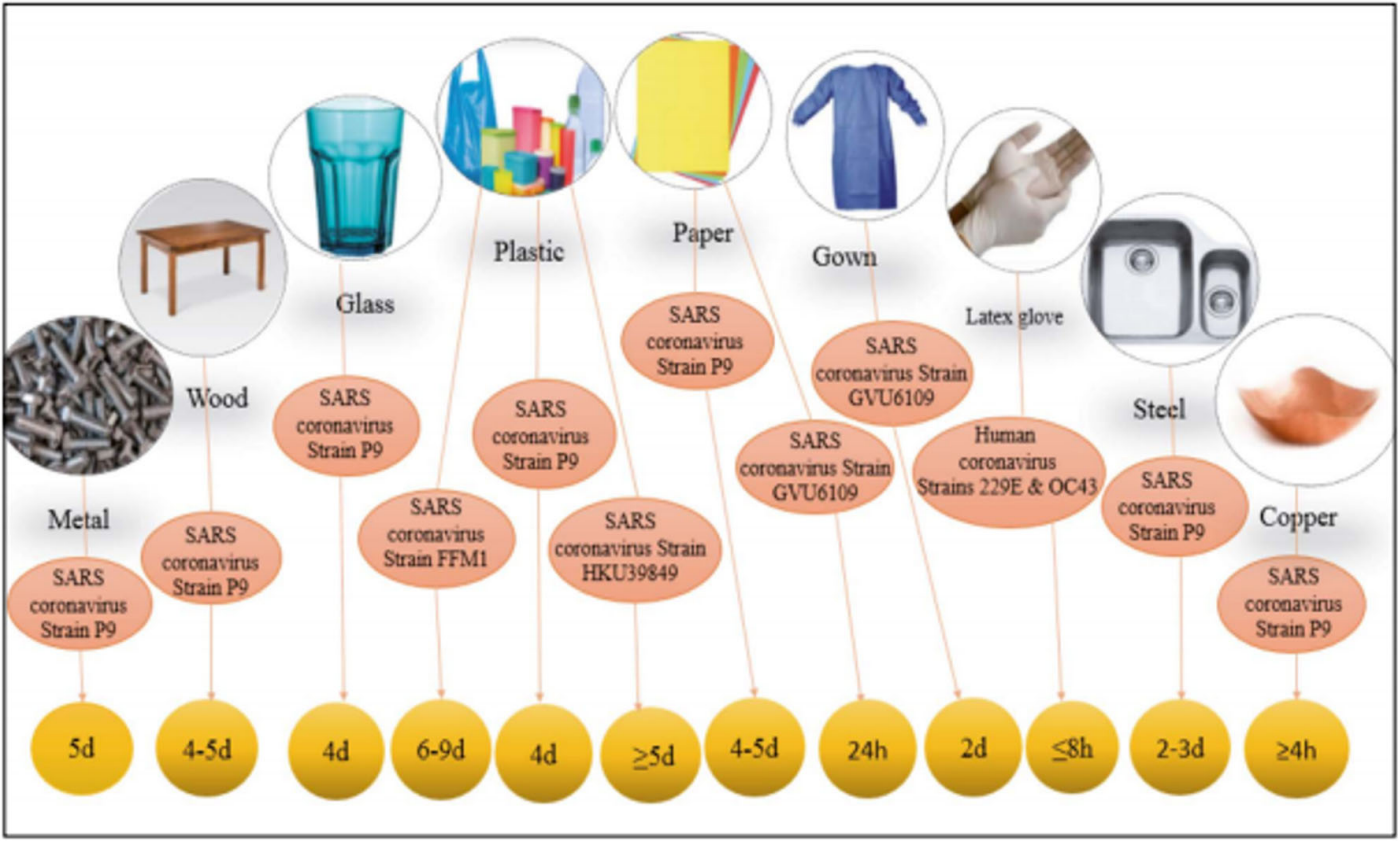
SARS-COV-2 persistence on surfaces (Fathizadeh et al. 2020)

The onset of serious illness will lead to death because of severe alveolar damage and progressive respiratory failure. Approximately 66, 580 cases and over 1524 fatalities were recorded as of 15 February 2020 (Xu et al. 2020). The World Health Organization (WHO) officially nominated the COVID-19 virus on 11 February 2020 (Guarner 2020). COVID-19, a readily transmitted disease that spreads mainly through respiratory droplets and then through infected surfaces, is known to cause the new coronavirus SARS-CoV-2(ECDC 2020). Recent experiments have found that this virus can sustain for hours to days on a variety of surfaces (Fig. 1)(Kampf et al. 2020; Van Doremalen et al. 2020). Kampf and his colleagues (Kampf et al. 2020) reported that human coronaviruses can live at room temperature for 9 days. This time can be up to 28 days for veterinary coronaviruses, and the surprising thing is that the survival of coronaviruses has become shorter with a temperature rise of 30° or more. This means that if the infected items are touched, the person will get the virus and become infected.

Another path of SARS-CoV-2 dissemination appears to be hand interaction with surfaces in an indirect manner polluted by infectious droplets that might help in the spread-out of the pandemic of COVID-19, which can then impact the lips, nose or eyes (Cai et al. 2020; Dhand and Li 2020; Subpiramaniyam 2020; Water 2020). The development of potential therapies and vaccines for coronavirus disease 2019 (COVID-19) has been a major focus of global research and the control of the COVID-19 pandemic remains the top priority globally. Given the current ineffectiveness of different techniques for preventing viral growth, the absence of focused therapies and the daily increase in cases, disinfection is an essential accessible tool to limit COVID-19 spread and directly battle SARS-CoV-2. SARS-CoV-2 is resistant to a wide range of disinfectants (Chin et al. 2020; EPA, 2020). To inactivate SARS-CoV-2, lipid solvents like ethanol, formaldehyde, isopropanol, sodium hypochlorite or hydrogen peroxide can be employed (Duarte and de Santana 2020).

Moreover, to prevent COVID-19 transmission and infection waves, disinfection of environments such as offices, healthcare settings, public transportation, markets, restaurants and auditoriums is required, taking into account viral presence, persistence, stability, viability and environmental influence on viral persistence. Cleaning, sanitization, disinfection and other ways to control the pandemic’s destructive impacts, on the other hand, should be subjected to change and development throughout time based on their negative effects on the environment and human health (Mukherjee et al. 2021). Hence, the current paper focuses on the positive applications, efficacy and negative repercussions of various disinfectants, as well as mitigation methods to counteract their detrimental impacts during the COVID-19 pandemic.

## Sustainability of coronavirus on different surfaces

Santarpia et al. (Santarpia et al. 2020) sampled various surfaces and personal belongings in the rooms of SARS-CoV-2-infected hospitalized patients, finding a positive COVID-19 virus average of more than 75%, measured by polymerase chain reaction experiments (PCR). Ye et al. (Ye et al. 2020) tested reverse transcription polymerase chain reaction (RT-PCR) on hospital environmental surfaces (printers, keyboards, medical equipment, etc.) (*N* = 626, where *N* is the number of samples), and 13.9% of the hospital artefacts were tested positive. While the load of SARS-CoV2 on inanimate surfaces at the time of the SARS-CoV-2 outbreak is unknown, disinfectants appear to reduce the load of viruses on the surfaces, mainly the patient-infected surfaces and the higher viral load region around the patient.

The WHO is recommending that adequate and efficient disinfection and environmental sanitation procedures be maintained. Surfaces in the environment should be properly washed with water and disinfectant. A significant type of hospital disinfectant such as sodium hypochlorite may be used (Asgharzadeh et al. 2007). With household disinfectants, it is important to disinfect and clean frequently accessed surfaces such as locks, toilets, tables, switches and sinks. For disinfection, different types of biocidal agents are used globally, including alcohols, sodium hypochlorite, hydrogen peroxide, chloride, etc. (Kampf et al. 2020). Disinfectants containing 62–71% ethanol or 0.1% sodium hypochlorite have been shown to reduce the risk of coronavirus accumulation on surfaces within a minute of contact (Kampf et al. 2020). After disinfection, cleaning should be carried out on contaminated surfaces. Examples of disinfectants and the time needed for their effect on the SARS coronavirus are given in Table 1.

**Table 1:** Examples of disinfectants and time needed for their effect in SARS coronavirus

**Virus**	**Biocidal agent**	**Reduction of viral infectivity (log**_**10**_**)**	**Exposure time**	**Reference**
SARS coronavirus strain FFM1	Ethanol 95%	≥ 5.5	30 s	Rabenau et al. (2005b)
	Ethanol 78%	≥ 5.0	30 s	Rabenau et al. (2005a)
	2-Propanol 75%	≥ 4.0	30 s	Siddharta et al. (2017)
	2-Propanol70%	≥ 3.3	30 s	Rabenau et al. (2005a)
	Formaldehyde1%	>3.0	2 min	Rabenau et al. (2005a)
	Glutardialdehyde 0.5%	>3.0	2 min	Rabenau et al. (2005a)
	Povidone iodine 0.23%	≥4.4	15 s	Eggers et al. (2018)
	Sodium hypochlorite 0.1%	≥3.0	1 min	(Kampf et al. (2020)

## Toxic effect of disinfectants

The disease COVID-19 has emerged as a challenge to humankind. The governing agencies have been trying to use different chemicals for disinfecting various commercial spaces, streets and roads to prevent the spread of the disease (Nabi et al. 2020; Palmer et al. 2020). There is little information available on the impacts; these disinfectant sprays would have on humans and the environment. According to numerous studies, exposure to most frequently used disinfectant compounds, viz. quats, sodium hypochlorite, hydrogen peroxide, alcohol and glutaraldehyde, led to an increased risk of chronic obstructive pulmonary disease (COPD), asthma and eye irritation on health workers and individuals when used regularly (Casey et al. 2017; Dumas et al. 2019; Weinmann et al. 2019).

Chemical residues on a surface can become airborne, contributing to poor indoor air quality, which can be harmful to asthmatics, allergic or sensitive individuals. These residues contain compounds that can cause cancer, reproductive problems, respiratory problems, eye and skin irritation, CNS impairment, oxidative damage and other human health problems (Choi et al. 2020). Moreover, during rains, the disinfectants would be washed away and thereby contaminating our water bodies, soil and air. Both direct and indirect sewage effluents will ultimately wind up in lakes and rivers, posing a threat to aquatic ecosystems and wildlife. Increased chlorine disinfectant levels can cause direct damage to organisms by disintegrating cell walls or oxidizing proteins (Sedlak and von Gunten 2011).

Disinfection chemicals can create hazardous secondary by-products such as trihalomethanes or halo acetic acids when they interact with other materials (Sedlak and von Gunten 2011). Aquatic creatures are found to be extremely harmful to these by-products(Liu and Zhang 2014). Hence, the same disinfectants could turn pollutants at a certain stage. Therefore, there is a need to discuss the negative effects of these disinfectants on humans and their surroundings. Several experts have alarmed environmental pollution concerns given the extensive and irresponsible spray of disinfectants amid the coronavirus outbreak. In India, the disinfectants have been extensively sprayed on buildings, streets and roads that have stirred widespread concerns of soil and water pollution.

## Role of chemical disinfectants in reducing COVID-19 outbreak

Chemical disinfectants have a low effective concentration, quick action, consistent efficiency and a wide sterilization range. This consumes bacteria spores as well as microorganisms. Since sodium hypochlorite, calcium hypochlorite and chlorine dioxide are non-corrosive to products, odourless, tasteless, colourless, inflammable, clean and easily soluble in water, they are commonly used. These, on the other hand, are resistant to physical and chemical causes, possessing low toxicity and no residual threat after disinfection. The commonly used disinfectants in India are either alcohol-based or poisonous chlorine-based disinfectants. Alcohol-based disinfectants are safe to use on the human body. However, these should be used with safety due to their volatile and flammable nature. Some of the chemical disinfectants used to prevent COVID-19 are discussed in below sections.

### Sodium hypochlorite

Sodium hypochlorite is the most commonly used disinfectant sprayed commonly in public places. Sodium hypochlorite has been historically used as a bleaching agent, but given its special properties, it has found widespread application in agriculture, food industries, waste disposal and pharma industries. Disinfectants like sodium hypochlorite are now being extensively used in preventing the coronavirus disease spread. When used at low concentrations, sodium hypochlorite has a negligible effect on the environment. The spraying of high concentrations and vast amounts of disinfectants, however, can be hazardous to human beings and the environment. Uncontrolled and widespread spraying of disinfectants can have a negative environmental impact. Because of sodium hypochlorite exposure, human beings will suffer many health effects. The lethal human (adult) dose of NaOCl was stated to be about 200 mL of a solution with 3–6% AvCI (Marrubini et al. 1987.

Due to sodium hypochlorite inhalation, exposed individuals may experience extreme coughing and throat pain. The aerosols created by the sodium hypochlorite spray can cause irritation to the respiratory tracks of living organisms including humans. Sodium hypochlorite can cause redness in the eyes and irritation when in contact with the skin. Nausea, vomiting and burning mouth pain are common gastrointestinal symptoms caused by household bleach containing up to 6% sodium hypochlorite (Racioppi et al. 1994). A 31-year-old man injected 0.3-mL sodium hypochlorite (Clorox@R) at a concentration of 5.25 % and suffered with acute left-side chest pain and numerous vomiting events in the right and left antecubital vein (Morgan 1992). Gnemi (1991) studied the acute eye inflammation effects of 0.1 mL or 0.11% NaOCl in rabbits. Sodium hypochlorite is also toxic to living aquatic species.

### Chlorine

Chlorine-dependent disinfectants create a completely new complex of very poorly researched pollutants. The acute toxicity of chlorine to aquatic species is high. Several toxicity levels for some aquatic organisms are less than or equivalent to 1 mg/L (AQUIRE 1994). Exposure to chlorinated water bodies has been linked to oral cavity papillomas in fish (Program 1992). Scientists have expressed concerns over the possible formation of secondary products because of a reaction between chlorine by-products and other naturally occurring substances. When in contact with ammonium salts, it becomes toxic and mutagenic. The by-products of these reactions can be carcinogenic. Scientists have reported more than 600 disinfection by-products, though the Environmental Protection Agency (EPA) regulates only (Richardson 2003). The regulated compounds include five haloacetic acids and four trihalomethanes which together are linked to a variety of adverse health effects like birth defects (Porter et al. 2005), cancer (Villanueva et al. 2007) and an increased incidence of miscarriage (Waller et al. 1998).

### Alcohol

Disinfectants containing 62–71% ethanol have been shown to eliminate coronavirus infection on surfaces in as little as 1 min (Fathizadeh et al. 2020). For instance, to disinfect the hands of trauma patients, a cleaning biocide kit with a gauze pad soaked in ethanol 62 to71% for at least 1 min may be used (De Vitis et al. 2020). Both switches and mice were disinfected twice a day with either 75% ethanol or disposable disinfecting wipes. In addition, after regular therapies, all spaces were disinfected by cleaning all surfaces with 75% ethanol (Wei et al. 2020). In oxygen therapy and respiratory treatment services, the surfaces of the respirator are disinfected with 75% alcohol every day (Members et al. 2020. Since completing their professional duties in a radiation oncology facility, Chen et al. (2020) propose that medical personnel’s masks and often touched things such as personal medical services, computer equipment, notebooks and other products should be replaced and disinfected with a 75 % alcohol disinfectant(Chen et al. 2020).

### Hydrogen peroxide (H_2_O_2_)

Hydroxyl free radicals are damaged by hydrogen peroxide solution (Abramowicz and Basseal). According to Zhang et al. (2020), in a critical care echocardiography centre, device probes were disinfected with H_2_O_2_ for each scan. In the endoscopy ward, H_2_O_2_ can also be used to clean ultrasound probes (Repici et al. 2020). The action time of H_2_O_2_ in radiotherapy centres is 30–60 min (Palmer et al. 2020). According to De Vitis et al. (2020), disinfect the hands of trauma victims, and use a washing biocide kit with a gauze pad soaked in 0.5% hydrogen peroxide for at least 1 min. To minimize the risk of bacterial infection on exposed fractures or unclean injuries, other packing is applied for at least 2 min with H_2_O_2_ wipes, followed by cleaning with a minimum of 1 L of 0.9% saline solution.

During tracheal intubation, the first layer of anaesthesiologists’ gloves should be disinfected with H_2_O_2_ solution, the outer layer gloves should be removed, and the inner gloves should be disinfected with H_2_O_2_ solution again, according to He et al. (2020). To clean the internal inhalation circuit during perioperative infection management, vaporized hydrogen peroxide was used to remove any residual pathogenic agents such as COVID-19(Li et al. 2020b). In the special channel used for suspected patients in the medical imaging clinic, H_2_O_2_ sprayed by an air sterilizer is used for infection control (Zhao et al. 2020). The air in the burn ward must be disinfected three times a day with H_2_O_2_ and acid peroxide, which can be used for ultra-low capacity spray disinfection (Li et al. 2020a). As a result, Table 2 contains a list of the WHO recommended disinfectants (Subpiramaniyam 2020).

**Table 2:** The World Health Organization (WHO) has published a list of recommended biocides that can be used to combat SARS-CoV-2(Subpiramaniyam 2020)

**Disinfectants**	**Concentration**	**Effectiveness**
Hydrogen peroxide	≥0.5% (5000 mg/L)	Highly effective
Sodium hypochlorite	0.1% (1000 mg/L) for general environmental disinfection and 0.5% (10,000 mg/L) for	Highly effective
Ethanol	62–71%	Highly effective
Phenolic compounds	According to the manufacturer’s recommendations	Highly effective
Ammonium compounds	According to the manufacturer’s recommendations	Highly effective
Benzalkonium chloride	0.05–0.2%	Less effective
Chlorhexidine digluconate	0.02%	Less effective

## Effects of disinfectants on living organisms

In the last decade, biocides have been scrutinized after it appeared that they could have a similar effect on surface waters as chemicals coming from industrial surface runoff (Wittmer et al. 2010). The risk quotient (RQ) is the ratio of a point estimate of toxicity to a point estimate of effects and hazard quotient (HQ) is the ratio of chemical contaminant concentration to a selected screening benchmark which is often used to measure the environmental risk of biocides in the aquatic ecosystem (Suter II 2016; Walker et al. 2017). RQ0.01 (very low), 0.01 RQ0.1 (low), 0.1RQ1 (medium) and RQ >1 (high) were the levels of environmental risk in terms of RQ values (Tato et al. 2018). HQ 1 (no substantial risk), 1 HQ 10 (small possible effects), 10 HQ 100 (significant potential adverse effects) and HQ >100 (adverse effects) were the criteria for HQ values. The RQ values are utilized to evaluate the impact of the biocide triclosan found in Santos Bay sediments on a sea urchin (Lytechinus variegatus) and a bivalve (Perna perna) (Pusceddu et al. 2018).

In an effort to reduce the transmission of coronavirus disease in 2019, disinfection affects aquatic ecosystems (COVID-19). In both the indoor and outdoor areas, China has used chlorine disinfectants. China has given at least two thousand tonnes of disinfectants in Wuhan City alone to minimize the potential of SARS-CoV-2, the virus that causes COVID-19, to survive. Open runoff and secondary drainage waste potentially end up endangering aquatic ecosystems in lakes and waterways (China Ministry of Ecology and Environment 2020) (Sedlak and von Gunten 2011). Chlorine disinfectants damage marine plants and animals in two respects. Firstly, by damaging their cell walls or oxidizing their proteins, chlorine can directly kill organisms (Sedlak and von Gunten 2011). Second, to form damaging compounds, the disinfectants contain chemicals that may bind with other materials.

Dissolved organic matter is exceptionally large in surface water (Baker 1994), which may allow for the synthetization of disinfectant by-products such as trihalomethane or haloacetate acids, for example (Sedlak and von Gunten 2011). It has been shown that these by-products are especially harmful to aquatic ecosystems (Liu and Zhang 2014). In addition, nitrogen, chloramine-forming or N-nitroso-dimethylamine-forming disinfectants may be mixed, all of which have been recognized as carcinogens (Krytopoulos 1979). As COVID-19 spreads across the world, increased disinfectant usage could result in secondary disasters in aquatic ecosystems all over the world. India began spraying disinfectants on numerous commercial and residential structures on both sides of the road, especially in urban/suburban areas, including metro cities. The disinfectant is sodium hypochlorite (NaOCl), an alkaline solution containing NaOCl. Surprisingly, it was even sprayed on individuals. Several disinfectant tunnels were set up in various locations, and people were asked to walk into them. People, including girls, were sprayed as they moved from one part of the country to another, according to reports reported in national and regional newspapers. Later, the Directorate General of Health Services of India’s Ministry of Health and Family Welfare released a warning against spraying the disinfectant on citizens.

However, large-scale NaOCl spraying continues over a number of officials, residential and industrial houses, parks, open fields, markets, stores, road transport and railways, among other places. The concentration of hypochlorite solution used is the main source of concern. Since there are no administrative/regulatory boards that have set and controlled the concentration, it varies widely. According to a survey (Chatterjee 2020), 5–10 % NaOCl solutions are used in the majority of the country; however, highly concentrated solutions (> 10 %) are still used in some cities. We urge China’s and other affected countries’ governments to conduct marine biodiversity integrity surveys before and after the pandemic. It would shield animals and people from any health risks posed by contaminated water.

The use of these disinfectants can be justified to some degree, given the severity of the COVID epidemic, but disinfectants need to be used cautiously when taking into account their long-term effects on humans and their environment. It is therefore important to control the widespread use of disinfectants on human beings. On 18 April 2020, the Union Ministry of Health released a notice against the spraying of disinfectants on people for the management of COVID-19, claiming that they were physically and psychologically dangerous. Even if a person is potentially exposed to the COVID-19 virus, as the ministry has said, the spraying of chemical disinfectants into the human body will not kill the virus. It also added that no empirical evidence exists to indicate that they are effective even inefficiently disinfecting the outer clothing/body. Spray tunnels are used extensively to spray disinfectants on humans.

Some researchers have revealed significant disadvantages of spray tunnels for disinfection. On soft surfaces like clothes, researchers have suggested their lack of quality. It would not be substantially safe against virus traces on the individual’s skin or clothes. They can also contribute to health and safety hazards. Sodium hypochlorite is a corrosive material, so employees need to be supplied with adequate equipment and accessories for eyes, skin and respiratory protection. Tiny droplets (< 20 μm) are created by powered spray systems that, in addition to contact with the eyes and skin, present a serious risk of inhalation by spraying. It can cause deep lung tissue inhalation, leading to negative health effects. No disinfectant has been tested for use by the general public through these methods. EPA continues to provide valuable information on surface disinfectant materials that can be used to protect people’s health during the COVID-19 public health emergency. There are a wide variety of excellent EPA-certified disinfectants that can provide several roles used mostly for disinfection in healthcare environments around the world for coronavirus degradation (Kampf et al. 2020).

Due to the COVID-19 pandemic, the manufacture, use and disposal of personal protective equipment (PPE), such as face masks, rubber gloves and disinfectant wipes, which are often made of single-use plastic, has increased exponentially (Ammendolia et al. 2020). The widespread use of these products has polluted the environment and put pressure on municipalities to collect and dispose of potentially contagious PPE properly. There have been little systematic surveillance attempts to measure the pattern of poorly disposed of PPE debris, which causes soil problems. Air disinfectants are chemical agents that can kill microorganisms that are trapped in the air. Uncontrolled and widespread spraying of disinfectants have degraded the quality of life and have caused allergies to people (Ammendolia et al. 2020).

## Selection and use of disinfectants against coronavirus

No great disinfectants or ideal ones are available so far. The active chemical disinfectants are described in Table 2 that are thought to be used against coronaviruses. Almost all chemical disinfectants, whether natural or synthetic, contain some kind of soap, oil or surfactant. Quaternary ammonium (Quats) is the active ingredient of some disinfectants; among them are hydrogen peroxide, peroxyacetic acid, ammonia, isopropanol, sodium hypochlorite, octatonic acid, phenolic acid, lactic acid and glycolic acid (Table 3). A specific item is intended for a certain reason in certain circumstances and must be treated in a certain way. The mark can then be carefully read to ensure that the appropriate content is used for the intended purpose and that the application is completed successfully.

**Table 3:** List of product ingredients certified by the EPA against SARS-CoV-2(Rai et al. 2020)

**Active ingredients**	**Formulation type**	**Effective contact time (min)**
Sodium hypochlorite	Dilutable	10
Hydrogen peroxide	Dilutable	10
Hydrogen peroxide	Dilutable	5
Peroxyacetic acid	Dilutable	1
Phenolic	Dilutable	10
Octanoic acid	Dilutable	2
Citric acid	RTU	1
Hypochlorous acid	RTU	10
Ammonium carbonate	RTU	6
Glycolic acid	Impregnated materials	10
Isopropanol	Wipe	0.5

In addition, cleaners and disinfectants must be used with caution to reduce risks. Otherwise, disinfectants may cause various forms of toxicity if they are mishandled, stored improperly or used often. In the USA, 60% of respondents said they cleaned or disinfected their homes more often than they had in the previous months. In the USA, approximately 19% of food products (such as fruits and vegetables) are bleached. Although household cleaning and disinfectant items are used 18% on hands or clothing, using a washing mist on the body or disinfectant spray is used 10%. Furthermore, the risk of inhaling vapours from household cleaners is 6%, and the risk of swallowing or gargling liquid bleach fluids, soapy water or other cleaning and disinfectant solutions is around 4% each (Gharpure et al. 2020).

Although limited information is available on the environmental impacts of these chemicals, care should be taken while spraying them for disinfection purposes. The government agencies should also have a check on the excessive spray of these chemicals for disinfecting surfaces. They should also consider other safe and biodegradable disinfectants for use in densely populated areas. Extensive research is also needed to evaluate the potential impacts of excessive use of disinfectants on living organisms and their surrounding environment.

## Safety issues and health concerns

**Fig. 2:**
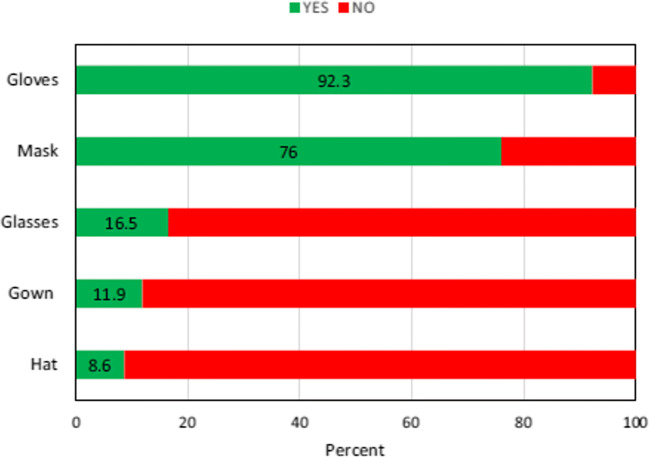
The PPE used by participants when the surfaces are disinfected (Dindarloo et al. 2020)

**Fig. 3:**
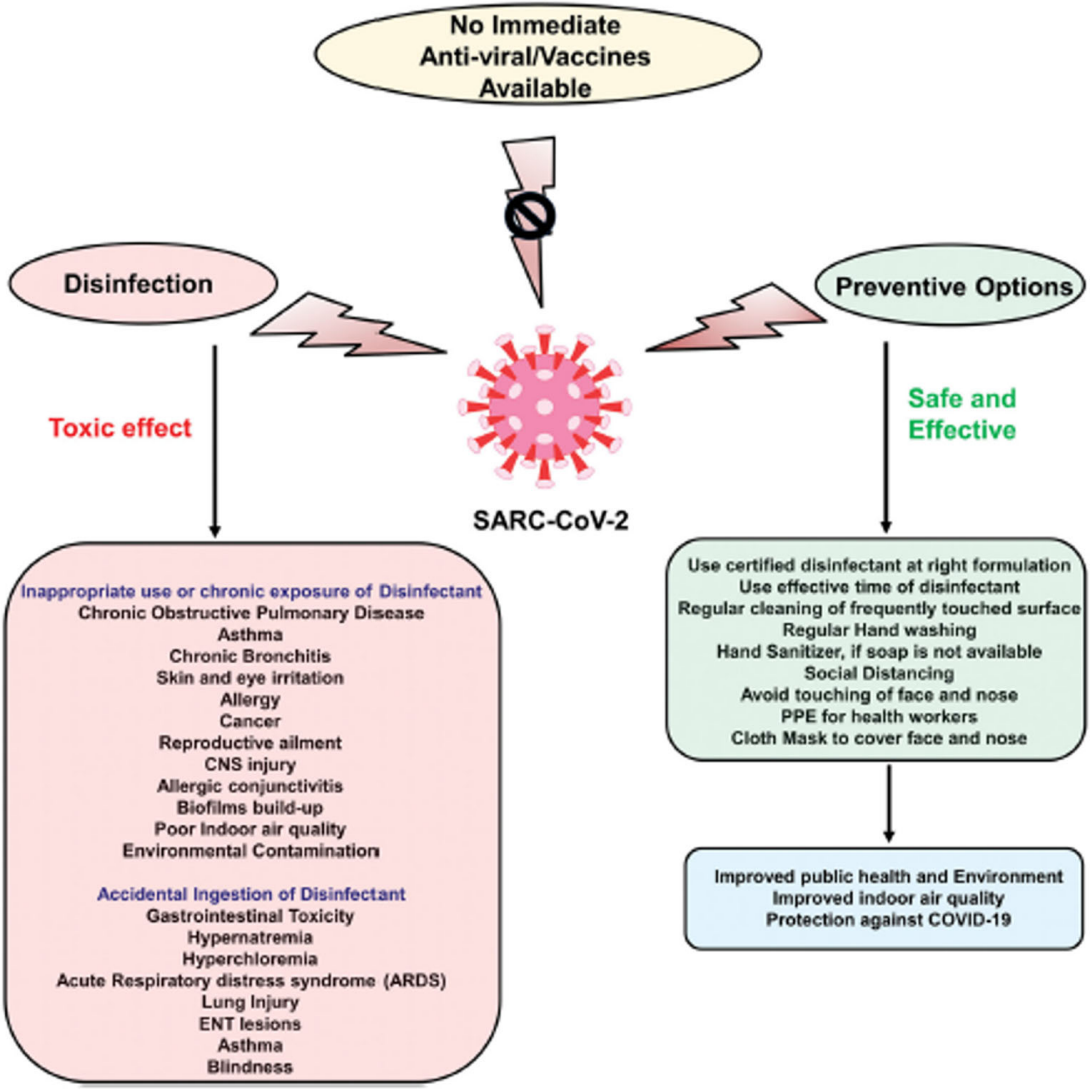
A description of the toxic effects of excessive use of disinfectants and healthy alternatives against SARS-CoV-2 infection is shown in the schematic representation (Rai et al. 2020)

One of the conditions for using surface disinfectants is the use of personal protective equipment (PPE), such as masks, gloves, hat, face shield, apron and goggles (Organization 2020; Ranney et al. 2020). Of people, 47.50% did not use any personal protective devices by using disinfectants on surfaces that risk their health. The percentage of different PPEs that users utilize is as presented in Fig. 2. Currently, there are various vaccines available at the moment. Irrespective of that, we should integrate hand hygiene and safe methods of disinfection into our everyday routine. We could maintain our well-being, enhance the quality of indoor air and protect the outdoor climate from COVID-19 by introducing these preventive measures (Fig. 3).

## Conclusion

The regulatory authorities have tried to deter the spread of the disease by using various chemicals to disinfect multiple commercial spaces, roads, highways, etc. Spraying disinfectants is an effective way to eradicate pathogens that cause viral diseases and human coronaviruses, which can stay infected on inanimate surfaces for days. There are clinically based recommendations for the prudent selection and usage of disinfectants in clinics, labs and homes that take into account their effectiveness, comfort and health hazards. However, there are no equivalent guidelines or monitoring systems for the systemic use of disinfectants currently being used in urban settings to fight these diseases. The rapid pace of research and development of several novel disinfectants against COVID-19 offers promise for the production of reliable, potent and convenient disinfectants that are cheap to everybody and accessible in a variety of locations with minimal or no harm to health and the environment. However, cautionary and preventive steps should be taken while using disinfectants. The increased usage of disinfectants necessitates an immediate environmental effect evaluation. To decrease the negative effects on individuals and the environment, clear and comprehensive disinfectant application rules are also required at the regional, national and international levels.

## Data Availability

Not applicable.
